# Supplementary material for: Supercurrent diode effect and magnetochiral anisotropy in few-layer NbSe2
Source: Nat Commun. 2022 Jul 23;13:4266. doi: 10.1038/s41467-022-31954-5 (PMC9308774; doi:10.1038/s41467-022-31954-5)
Supplement: Supplementary file 1 — Supplementary Information [file 41467_2022_31954_MOESM1_ESM.pdf]

Supplementary Information: Supercurrent diode effect and magnetochiral anisotropy in few-layer $NbSe_2$

Lorenz Bauriedl,[1] Christian Bäuml,[1] Lorenz Fuchs,[1] Christian Baumgartner,[1] Nicolas Paulik,[1] Jonas M. Bauer,[1] Kai-Qiang Lin,[1] John M. Lupton,[1] Takashi Taniguchi,[2] Kenji Watanabe,[2] Christoph Strunk,[1] and Nicola Paradiso[1,*]

[1]*Institut für Experimentelle und Angewandte Physik, University of Regensburg, Regensburg, Germany*
[2]*International Center for Materials Nanoarchitectonics, National Institute for Materials Science, Tsukuba, Japan*

SUPPLEMENTARY NOTE 1: ADDITIONAL MEASUREMENTS ON SAMPLE G

In this section we present additional experimental data from sample G. Supplementary Figure **1a** shows the raw data (namely, without subtraction of -2.5 mT in B_z and 170 nA in current) used to generate the plot in Fig. 1**f** of the main text. For each value of B_z, ten IVs for positive bias and ten for negative bias where measured, whose critical current values are displayed in Supplementary Fig. **1a**. A certain distribution in the switching current is visible. Each data point in Fig.1**f** of the main text is obtained averaging over these points. Without the averaging, the scatter in $I_c^{\pm}$ would produce a larger scatter in Q, owing to the fact that this latter quantity is obtained from the difference between I_c^+ and $|I_c^-|$.

Supplementary Figure **1b** shows measurements of the rectification efficiency Q as a function of B_z for different B_z-sweep directions. As for panel **a**, here we neither subtracted instrumental offsets, nor averaged the data. We notice that, within the data scatter, there is no apparent effect of the sweep direction.

Finally, Supplementary Fig. **1c** shows a $R(T)$ curve as extracted from IV characteristics measured in a three-terminal configuration. Each data point is obtained by fitting the low bias part of the IV. The temperature independent value at low temperature ($R(0) \approx 650\ \Omega$) is the source contact resistance, which is unavoidable in a three-terminal measurement. From the $R(T)$ we deduce a $T_c = 4.0$ K at the constriction. As for the other samples, this value is smaller than the typical T_c for few-layer $NbSe_2$ (> 5 K), owing to the disorder introduced in the constriction by etching.

Offset removal in sample G. In Supplementary Fig. **2a** we show the same data as in Fig. 1**f** –namely, I_c^+ and $|I_c^-|$ for sample G– but, in this case, without removing the field offset (-2.5 mT) and the current offset (170 nA). The graph is nearly indistinguishable from that in Fig. 1**f**, indicating that the offset has only a minor impact. In particular, the current offset is about 1.5% of the maximum critical current. The resulting $Q(B_z)$ curve without offset removal is plotted in Supplementary Fig. **2b**. As a result of the offset, the curve appears slightly shifted (both horizontally and vertically) compared to Fig. 1**g** of the main text. Note that the offset is relatively small, comparable with the width of the switching current distribution discussed above: its effect becomes visible only in the averaged $Q(B_z)$ curves, as the one shown in Fig. 1**g** or Supplementary Fig. **2b**.

A subtraction of $\delta B_z = -2.5$ mT and $\delta I_c = 170$ nA from the nominal values of, respectively, B_z and $I_c^{\pm}$[1], leads to a match of the $I_c^+(B_z)$ and $|I_c^-(-B_z)|$ curves, that is required by time-reversal symmetry. The overlapping curves are shown in Supplementary Fig. **2c**.

SUPPLEMENTARY NOTE 2: MEASUREMENTS ON SAMPLE B

In this section we discuss measurements on sample B. This sample (as well as samples D, E discussed below) was measured in the 1K cryostat with the magnetic field oriented nominally in-plane (and perpendicular to the current direction within the constriction). Owing to a misalignment (of typically a couple of degrees) the field has also a small but decisive component out-of-plane which produces the supercurrent rectification. Clearly, to obtain a B_z field of tens of mT, B_{ip} needs to be several teslas. On the other hand, owing to the characteristics of Ising superconductors, an in-plane field of few teslas has a little effect on the critical current.

Supplementary Figure **3a** shows a magnified version without contour lines of the micrograph in Fig. 1**b** of the main text. Figures **3b**, **c** and **d** show IV-characteristics for an applied field of 0 T, 2 T and -2 T, respectively. This field magnitude corresponds roughly to the maximal rectification. Notice that the positive critical current for 2 T is clearly larger that that for 0 T: similar to sample G, sample B (as well as sample D and E, see below) shows an increase of the critical current with the field. for one polarity. Supplementary Figure **3e** shows the dependence of the positive (black) and negative (red) critical current on the magnetic field. Each curve contains points from two different measurements performed with different resolution and at slightly different temperature (high resolution: $T = 1.4$ K, circles; low resolution: $T = 1.6$ K, triangles). For better comparison, the critical current are displayed normalized to the zero field value. Finally, Supplementary Fig. **3f** shows the field dependence of the rectification efficiency $Q \equiv 2(I_c^+ - |I_c^-|)/(I_c^+ + |I_c^-|)$. Panels **b-f** are the corresponding ones of panels **c-g** in the Fig. 1 of the main text.

These measurements show that the qualitative behavior of sample B is similar to that of sample G. Owing to the unknown degree of misalignment, it is not possible to determine

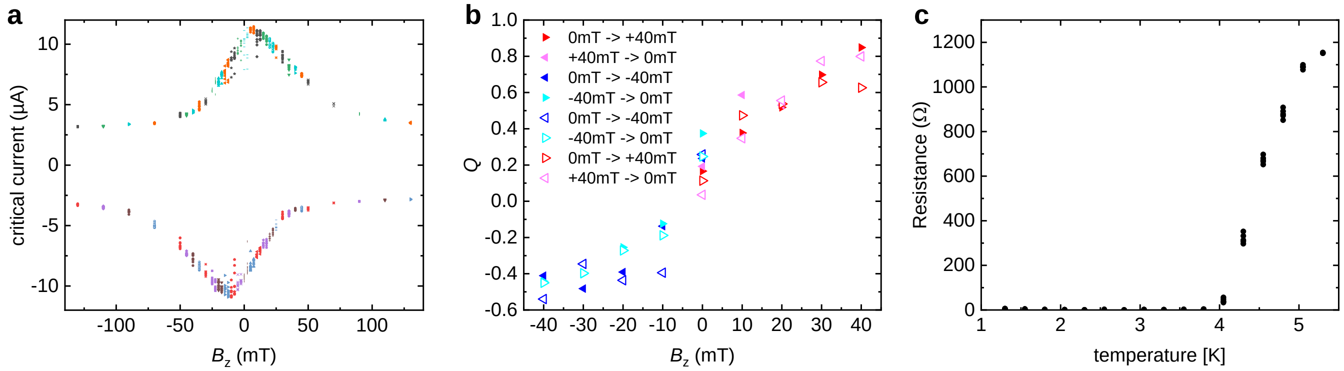

Supplementary Figure 1. **Additional measurements on sample G. a**, Positive and negative critical currents measured as a function of B_z in sample G. For each B_z value the different symbols correspond to different measured IVs. In this case, data are displayed as-measured, without instrumental offset subtraction. The graph highlights the significant spread in the switching currents. **b**, Supercurrent rectification efficiency Q measured as a function of B_z in sample G. The different symbols and colors refer to different B_z-sweep directions. **c**, Resistance measured in a three-terminal configuration in sample G. The resistance is deduced by fitting the low-bias part of IVs measured at different temperatures. A constant value of 650 Ω, corresponding to the source contact resistance, has been subtracted.

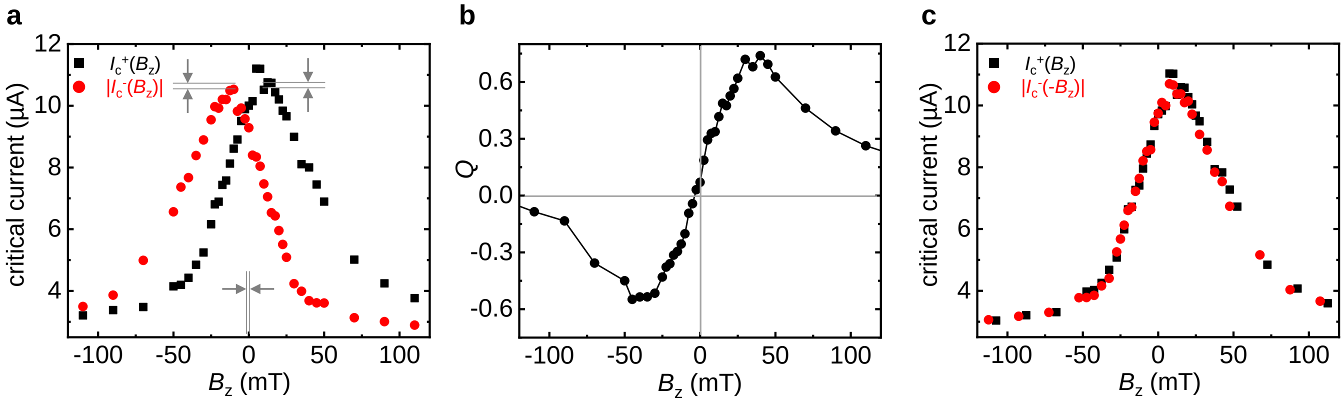

Supplementary Figure 2. **Magnetic field and current offset in sample G. a**, Positive and (absolute) negative critical current as a function of B_z in sample G, displayed without applying offset subtraction, in contrast to Fig. 1**f** of the main text. Each point corresponds to the average of ten measurements. Grey bars and arrows indicate the magnitude of the offsets. As a result of the offset subtraction the $I_c^+(B_z)$ curve (black) is shifted downwards, while $|I_c^-(B_z)|$ is shifted upwards, and both are shifted in the horizontal positive direction. **b**, Rectification efficiency Q as a function of B_z as deduced from panel **a**. In absence of offset subtraction the $Q(B_z)$ curve appears shifted upwards by 0.1 compared to the graph in Fig. 1**g**. **c**, Plot of $I_c^+(B_z)$ and $|I_c^-(-B_z)|$ after applying an offset $B_z^{\mathrm{off}} = -2.5$ mT and $I^{\mathrm{off}} = 170$ nA. This corresponds to Fig. 1**f** of the main text, with inversion of the abscissas for $|I_c^-|$.

neither $B_{\max,I_c}$ nor $B_{\max,Q}$. However, since by experience the misalignment is of the order of a couple of degrees, we estimate B_z to be in the range of several tens of mT, which is also in line with sample G and sample F.

SUPPLEMENTARY NOTE 3: SUMMARY DESCRIPTION OF THE OTHER SAMPLES

In this section we briefly describe other samples investigated within this project which have not yet been discussed so far, namely, samples A, C, D, E. The letter used to label the samples indicated the sequence of their fabrication (sample A first, sample G at last).

Sample A. In this sample, measured in 4-terminal configuration, the central constriction stayed normal all the way down to the base temperature of out 1K cryostat. This was likely caused by an unintentionally more aggressive reactive ion etching step, or by oxidation occurred after the lithographic fabrication of the constriction. Therefore, this sample shall not be further considered here.

Sample C. This sample has a different geometry (1 μm wide and 4 μm-long channel) and a different substrate (ceramic substrate for solid ion gating experiments) compared to all the others. It does not show a well-defined critical current: instead, it displays a series of phase slip lines, which some of the authors have studied in plain few-layer $NbSe_2$ crystals [2]. Owing to the peculiarities of this sample, we shall not consider it further.

Sample D. This sample (nominally identical to A, B, E, F).

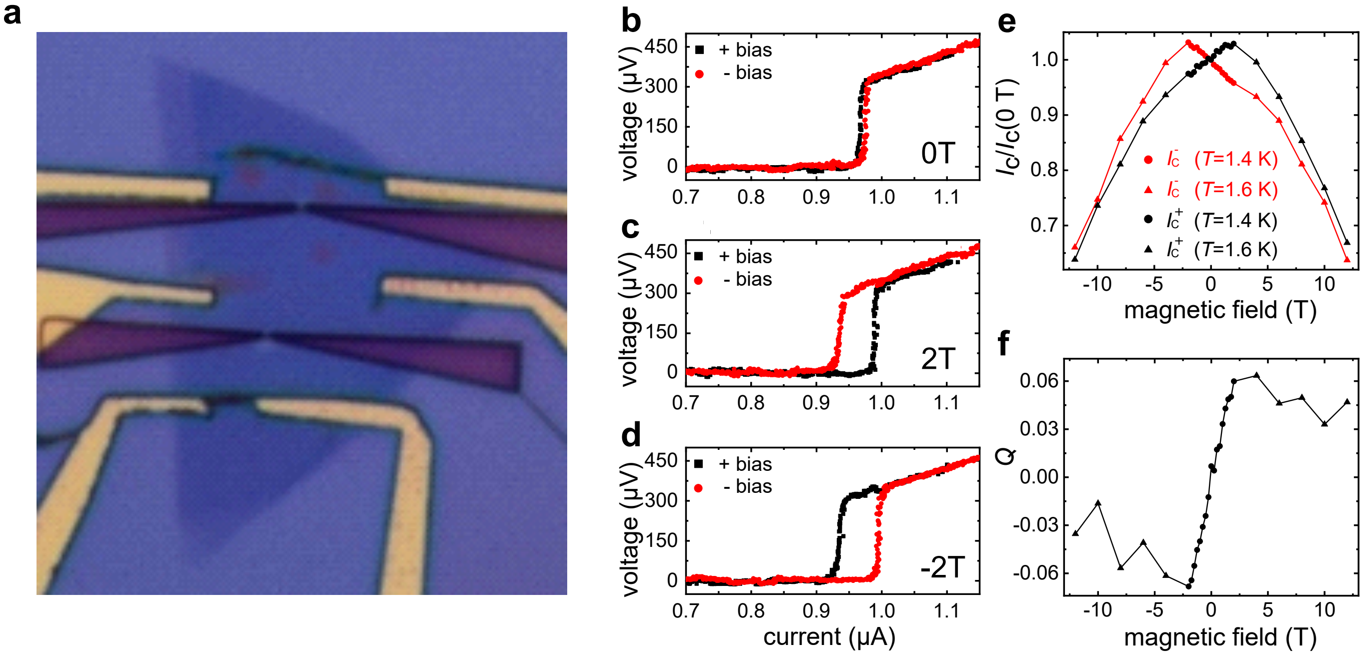

Supplementary Figure 3. **Measurements on sample B. a**, Optical microscope picture of sample B. **b**, Current-voltage characteristics (IVs) for opposite bias polarities (i.e., opposite current directions) measured in the absence of a magnetic field at $T = 1.4$ K on sample B. The sweep direction is always from zero to finite bias. **c**, Similar measurements, but in the presence of a magnetic field $B = 2$ T, mostly directed in-plane but with an important additional B_z component (see text). Notice the difference between the two critical currents. Importantly, the critical current for positive bias is larger than that in the absence of a field. **d**, Same as in-panel **c**, but with opposite field orientation. The role of the two bias polarities is now swapped. **e**, Critical current for positive (black) and negative (red) bias as a function of the magnetic field. We combine two series of measurements at 1.4 K (circles) and 1.6 K (triangles). All values are normalized to that at zero field. **f**, Supercurrent rectification efficiency $Q \equiv 2(I_c^+ - |I_c^-|)/(I_c^+ + |I_c^-|)$, plotted versus magnetic field.

The plot of the two critical currents $I_c^+(B)$ and $I_c^-(B)$ is similar to that in Fig. 1**f** of the main text. Similarly the plot of $Q(B)$ mirrors that in Fig. 1**g**. Critical current and supercurrent rectification efficiency measurements, reported in Supplementary Fig. **4a**, show a behavior similar to that observed in sample B, c.f. Fig. 1 of the main text. Also in this device the absolute value of the critical current increases with $|B|$, reaching a maximum for $|B| \simeq 0.4$ T. This value is smaller than that for sample B (about 2 T), most likely owing to a larger field misalignment in sample D. This sample shows the second-largest rectification efficiency: for $B = 1.5$ T, Q is as large as 33%, see Supplementary Fig. **4b**.

Sample E. The critical current of this device is very large. The I_c value, well above 100 μA, is close to the critical current for an entire flake and it is clearly incompatible with a width of few hundreds nanometers which is that of the constriction. The most likely interpretation is that, due to some undetected problems during the reactive ion etching step, the etching depth did not exceed that of the top hBN layer, leaving the $NbSe_2$ flake unaffected. As a consequence, the direction of the current is not well-defined as for the other samples. Despite this, we do observe a clear supercurrent diode effect, see Supplementary Fig. **4c-f**. From SHG measurements we deduce that the supercurrent direction within the constriction is nearly parallel (2° misalignment) to the armchair direction of the lattice, see next section.

We measured sample E in two cool-downs. In the first cool-down the applied field was mostly directed in-plane and parallel to the nominal constriction direction. In the second, the sample was rotated by 90° so that the field was still mostly directed in-plane, but perpendicular to the nominal constriction direction. The lack of a well-defined constriction makes it difficult to disentangle the effect of the different field components. Possibly because of that, the $I_c^{\pm}(B)$ and the $Q(B)$ plots appear asymmetric and distorted. It is important to remark that a supercurrent rectification is nevertheless observed, confirming the robustness of the diode effect in $NbSe_2$.

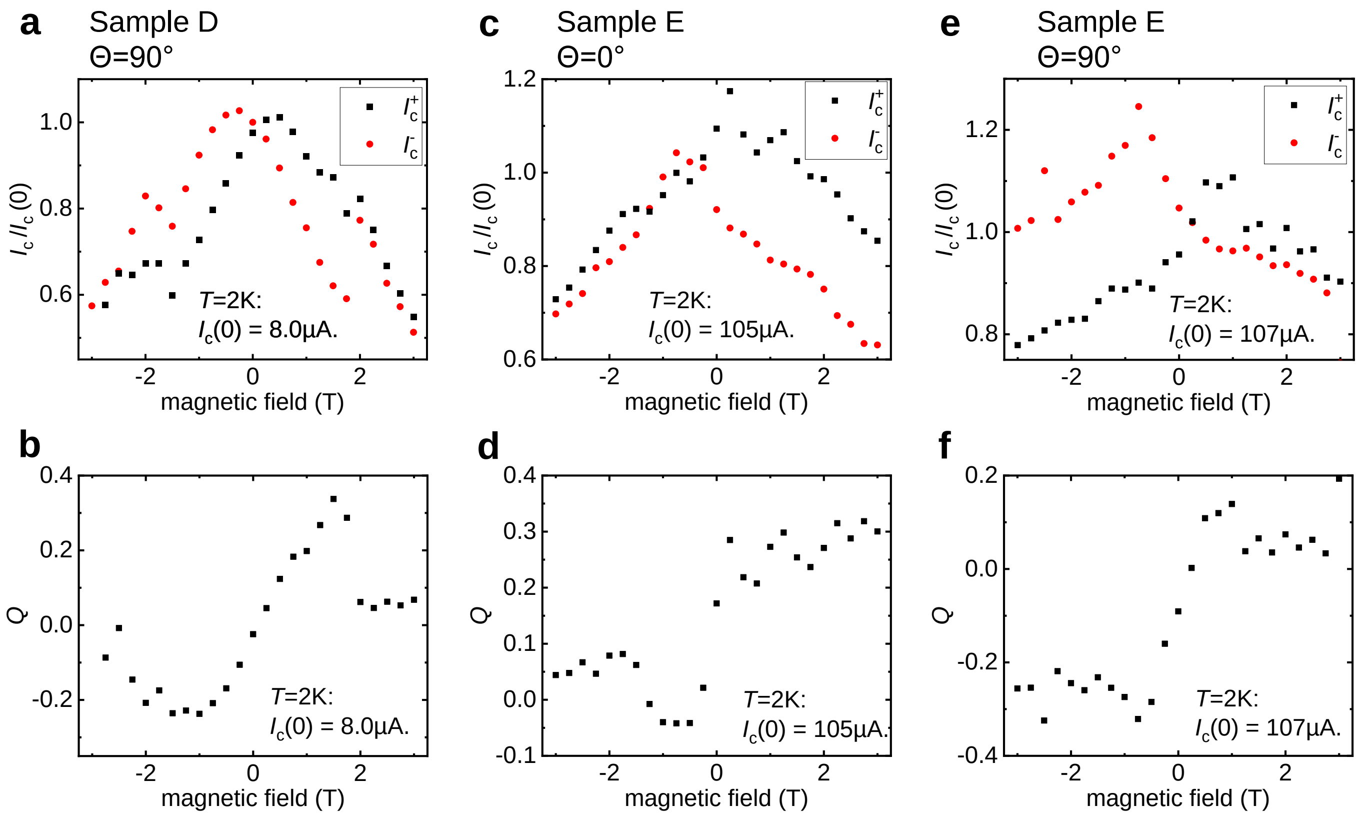

Supplementary Figure 4. **Critical current and rectification efficiency in other samples. a, c, e**, Magnetic field dependence of the critical current for positive (I_c^+) and negative (I_c^-) bias, measured in sample D (panel **a**), in sample E (panel **c** and **e**). The magnetic field is oriented mostly in-plane, with an important (but unknown) out-of-plane component. The in-plane field component is perpendicular ($\theta = 90^\circ$) to the current in **a** and **e**, while in panel **c** it is parallel to the current. **b, d, f** Magnetic field dependence of the rectification efficiency Q measured in sample D (panel **b**) and in sample E (panel **d** and **f**). Each graph is obtained from the data in the panel immediately above.

SUPPLEMENTARY NOTE 4: SECOND HARMONIC GENERATION MEASUREMENTS

To determine the armchair direction and layer number parity of the $NbSe_2$ crystal, we measure the co-polarized SHG intensity as a function of relative angle between laser polarization and crystal orientation [3–6] A Ti:sapphire laser (Spectra-Physics, Mai Tai XF, 80 MHz repetition rate, 80 fs pulse duration) at 800 nm was focused onto the $NbSe_2$ samples placed in the vacuum chamber using a microscope objective (Olympus, LUCPLFLN 40×) with numerical aperture of 0.6. The reflected SHG signal at 400 nm was collected with the same objective, filtered by a 680 nm short-pass filter (Semrock, FF01-680SP), dispersed in a spectrometer (Princeton Instruments, Acton SP2300) with a 150 grooves/mm grating, and detected by a CCD camera (Princeton Instruments, PIXIS 100). A linear polarizer was placed in front of the spectrometer to ensure acquisition of the signal parallel to the laser polarization. A 50:50 non-polarizing beam splitter was used to separate the incident path and signal detection path. An achromatic half-wave plate was placed between the beam splitter and the objective to change the relative angle between the crystal orientation and the laser polarization. The half-wave plate was rotated in 1° increments from 0° to 180° using a stepper motor, and the SHG intensity was recorded after each step. In general, a laser power of 1 mW and a single exposure time of 1 s were used for each measurement.

The polar plots in Supplementary Fig. **5** show the typical six-fold symmetry pattern representing the three-fold symmetry of the transition metal dichalcogenide crystals. The maximum (minimum) intensity direction corresponds to the armchair (zigzag) direction of the crystal. The light blue arrow indicates the axis of the constriction, i.e., the direction of the supercurrent in the transport measurements. The arrow is reproduced on the optical micrograph on the right side of each panel: there, the perpendicular dashed line crosses the arrow exactly at the position where the constriction is located. The yellow dot indicates the position where the corresponding SHG measurement indicated in the graph was performed. In the figure, panel **a**, **b**, **c**, and **d** refer to sample B, D, E, and F, respectively. The angle α between supercurrent and armchair direction is indicated in red on the top left of each panel. Only for sample G, the angle dependence is not reported: owing to its even number N of layers, the SHG signal is very small, except at the very edges. This sample is also very homogeneous, therefore it does not feature terraces with odd N which could allow us to perform an angle-resolved SHG measurements with a discernible signal. SHG measurements on this sample were nevertheless precious, since they confirmed its parity and demonstrated unambiguously that the supercurrent diode effect can be observed in crystals with both even and odd N.

From the *combination* of optical microscopy and SHG measurements, we deduce that samples B, D, E, F, G consist of 3, 3, 5, 3, and 2 layers, respectively. In fact, optical microscope pictures do not make it always possible an univocal determination of the layer number: a typical optical micrograph is typically compatible with two consecutive values of N.The parity determination provided by SHG eliminates the residual uncertainty in N.

Concerning the direction of the supercurrent with respect to the underlying lattice, in sample D and in sample E the vector $\vec{I}$ is almost perfectly aligned parallel to the armchair direction while in sample F is nearly parallel to the zig-zag direction. Finally, in sample B the orientation is intermediate, but closer to zig-zag.

We notice that the different magnitude of the supercurrent rectification factor among the samples does not seem to be correlated to the angle α. The same applies to the other peculiar phenomenon observed in our experiment, namely, the increase of the critical current with the magnetic field. This effect produces an opposite horizontal shift of the Λ-shaped $I_c^+(B)$ and $I_c^-(B)$ curves, see Fig. 1**f** of the main text and Supplementary Fig. **4a**-**c** here. The only sample where this effect is negligible (see Fig. 2**d** of the main text) is F, where $\vec{I}$ is oriented approximately along the zig-zag direction. On the other hand, in sample B the effect is particularly pronounced, despite an angle α relatively close to that for sample F.

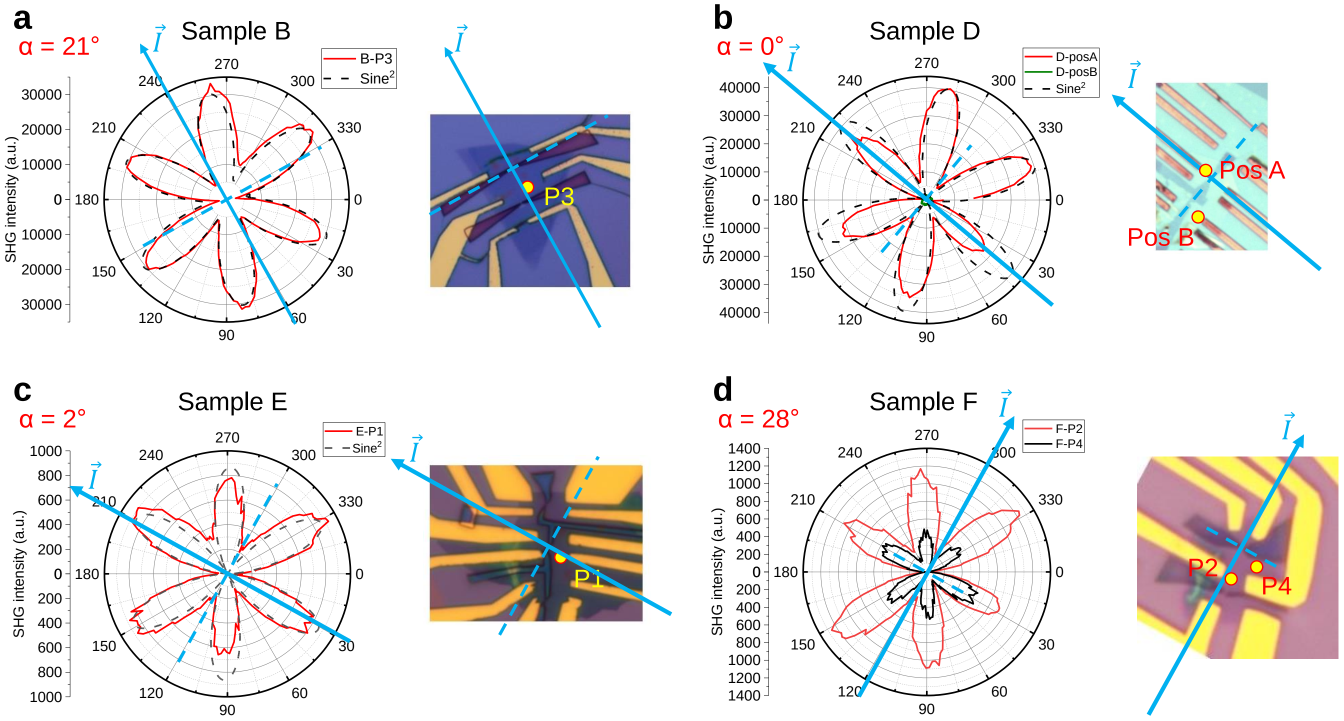

Supplementary Figure 5. **Second harmonic generation measurements.** **a**, Polar plot of the second harmonic generation (SHG, red curve) signal measured on sample B in the position indicated by the yellow dot (position P3) in the optical micrograph displayed on the right side. The dashed line in the graph shows a $\sin^2$ fit. The supercurrent direction (vector $\vec{I}$) is indicated on both sides by a light blue arrow. On the micrograph, the relevant constriction is located where the perpendicular dashed line crosses the arrow. The angle α between $\vec{I}$ and the armchair direction is indicated in red on the top left. **b**, The same for sample D. The red curve (pos A) corresponds to the 3-layer $NbSe_2$ area where the constriction is located. The weak signal shown by the green curve (pos B) corresponds to another area with even number of layers. **c**, The same for sample E. **d**, The same for sample F. The position P4 is closer to the constriction but both signals refer to the same layer number.

SUPPLEMENTARY NOTE 5: THE NEGLIGIBLE ROLE OF VORTICES

In 2D Rashba superconductors the supercurrent diode effect is driven by in-plane magnetic field, as shown by experiments [7, 8] and predicted by theory [9]. Therefore, the presence of vortices can be, in principle, eliminated by careful compensation of the out-of-plane component. In contrast, in $NbSe_2$ (and, more generally, in materials with valley-Zeeman spin-orbit interaction) it is precisely the out-of-plane field component that drives the supercurrent nonreciprocity, leading to the unavoidable presence of vortices. These might conceivably introduce spurious nonreciprocity, e.g. in the presence of an asymmetric barrier for entering/leaving the samples at the two edges of the constriction. However, the experimental evidence indicates that the presence of vortices does not play a significant role owing to the following arguments.

- We do not observe a measurable dissipation in the IVs until we reach the critical current, and this remains true up to fields at least as large as $B_{\max,Q}$. As an example, in Supplementary Fig. **6** we show IV characteristics for sample D (where 4-terminal IV-characteristics are available). In this case, as described above, the field is applied mainly in-plane, with an important out-of-plane component (of the order of several tens of mT). Notice that there is no measurable foot within a voltage scale of 500 nV and at the critical current the voltage emerges abruptly from the noise floor.

- If the diode effect were due to the asymmetry in the barrier for vortices entering/leaving the sample, the rectification would be extremely temperature dependent (following the temperature dependence of the penetration depth λ and thus the vortex barrier) while approaching T_c. Instead, the T-dependence in sample D and F is even nonmonotonic, and in general it does not follow the typical T-dependence of λ.

- If the vortex barrier played a significant role, one would expect a significant vortex trapping within the constriction, leading to hysteresis in the field sweep. Instead, as shown in Supplementary Fig. **1b**, the B_z-sweep direction is irrelevant.

- If the diode effect were due to vortices, the in-plane $\mathbf{B}_{ip}$ field would not make the effect of B_z asymmetric, as we observed in the experiments. *A fortiori*, the $\mathbf{B}_{ip}$ sign and orientation would be totally irrelevant, in contrast to results shown in Fig. 2 of the main text.

At the end of the review process for the present article, we became aware of the work by Hou *et al.* [10], who demonstrated a diode effect in plain V or Nb superconducting films in a out-of-plane field. The authors of Ref. [10] interpreted their observations with an asymmetric edge critical current density (i.e. asymmetric barrier for vortex entry) combined with transport and Meissner currents. To observe a bias polarity-dependent current increase, the authors consider the possibility that vortices enter from *either* sides of the samples. Their experimental results indicate that great care must be taken when interpreting the origin of asymmetric critical current in an out-of-plane fields. For what concerns our work, we believe that the mechanisms highlighted in Ref. [10] do not apply to the $NbSe_2$ constrictions here discussed owing to the following reasons:

- the width $w = 250$ nm of the constriction is comparable to the penetration depth of $NbSe_2$. Therefore the impact of the screening currents is highly suppressed compared to larger films as those discussed in Ref. [10].

- Vortex entry in few-layer $NbSe_2$ samples does not imply an immediate switch to normal current transport [2]. Instead, a substantial critical current is visible up to B_{c2}, a field much larger than the Bean-Livingston barrier. For large out-of-plane fields (typically of the order of 1 T, i.e., larger than those applied in the experiments reported in the main text) a nonlinear foot appears in the IV characteristics, which is due to vortex creep. This foot does not affect the measured I_c, which is the bias at which the resistance abruptly switches to the normal value.

- In Ref. [10] the critical current asymmetry peaks precisely when I_c^+ or I_c^- reaches its maximum. This is not the case in our experiments, see e.g. results on sample G, D, and F. In particular sample F shows a maximum critical current at *zero* out-of-plane field. What produces the critical current asymmetry in our samples is the different slope in the B_z dependence of I_c^+ and I_c^-, see Fig. 2 of the main text.

SUPPLEMENTARY NOTE 6: MAGNETIC FIELD MISALIGNMENT

As discussed in the main text, the alignment of the magnetic fields with respect to the sample surface is crucial for the interpretation of the data. In fact, the perturbing effect of $\vec{B}_{ip}$ is noticeable for fields of the order of teslas. On the other hand, a couple of degrees of field misalignment would produce a spurious out-of-plane field of the order of tens of milliteslas, more than sufficient to induce a sizeable diode effect.

The sample misalignment is due to two factors. First, the sample rotation axis might be not perfectly perpendicular to $\vec{B}_{ip}$. Second, the sample surface might not be perfectly perpendicular to the rotation axis. Owing to both misalignment effects (each of the order of about 1 or 2 degrees) the angle between $\vec{B}_{ip}$ and the sample surface varies as a function of θ with 2π-periodicity. As a consequence, measurements with B_z at fixed B_{ip} will be affected by an offset proportional to B_{ip}, with constant of proportionality being a 2π-periodic function of θ.

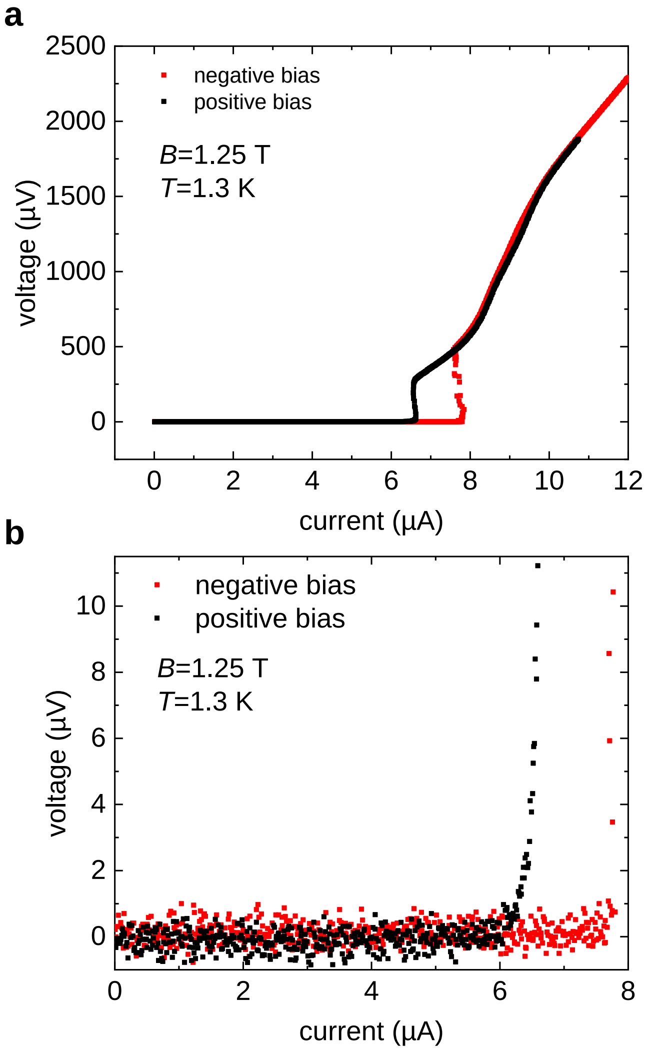

Supplementary Figure 6. **Emergence of dissipation in sample D.** **a**, Current-voltage characteristics measured on sample D. The black (red) symbols refer to the positive (negative) bias polarity. **b**, Zoom-in on a voltage scale of ≈ 10 µV. It is evident that dissipation emerges abruptly from the noise floor at the critical current value, without showing the typical smooth foot produced by vortex creep.

The determination of such an offset (and thus of the misalignment) can be easily achieved by measuring the critical current (either I_c^+ or I_c^-) as a function of the nominal B_z and B_{ip} fields. For an Ising superconductor the application of a few teslas in-plane field has little impact on the critical current, while out-of-plane fields of even a few tens of milliteslas have a noticeable effect. Therefore, if we plot, as in Supplementary Fig. **7**, $I_c^+(B_z,B_{ip})$, the $B_z(B_{ip})$ offset can be determined from the local maximum. For a given B_{ip} the maximum of $I_c^+(B_z)$ must occur when the *effective* B_z field is zero. This condition allows us to find, for that value of θ, the additional B_z introduced by B_{ip}. We deduce that $B_z = B_{z,\mathrm{nom}} + \kappa B_{ip}$, where B_z is the effective out-of-plane field (used in the main text, see Fig. 2a-c), $B_{z,\mathrm{nom}}$ is the nominally applied out-of-plane field, and κ is a coefficient such that $\kappa(90°) = 0.0343$, $\kappa(0°) = 0.0218$, and $\kappa(-90°) = -0.00256$. Approximately, from measurements at other intermediate angles θ, we found that $\kappa(\theta) \approx 0.015 + 0.019\sin(\theta - 0.30)$, with θ expressed in radians.

SUPPLEMENTARY NOTE 7: RESISTANCE AND CRITICAL CURRENT VERSUS TEMPERATURE

The graphs in Supplementary Fig. **8a**,**b** show the zero-bias resistance versus temperature for the constriction in sample D and F, respectively. Measurements on sample D were performed in 4-terminal, by combining an AC (lock-in amplifier) and a DC (digital-to-analog converter) voltage source, and by measuring the output current with a current amplifier and the voltage drop with a differential voltage amplifier. $R(T)$ curves are obtained from the zero-bias differential resistance measured with the lock-in amplifier, while the critical current is obtained from the DC IVs. Measurements on sample F were performed in 3-terminal configuration (since one of the 4 contacts did not work), using only DC excitation. $R(T)$ data are obtained from the low-bias slope of the IVs, upon subtraction of the constant contact resistance of 413 Ω. The temperature for which the resistance emerges from the noise floor of the deep superconducting state is 3.68 K in sample D and 2.2 K in sample F.

The graph in Supplementary Fig. **8c** shows the temperature dependence of the positive and negative critical current. We stress that, as throughout this work, the IVs from which we determine I_c are always swept from zero bias to finite (either positive or negative) bias, in order to eliminate any possible heating effect. The full symbols in Supplementary Fig. **8c** refer to critical current values deduced from the threshold condition $V = V_{thres} \equiv 100$ µeV. The empty symbols refer to critical current values deduced by extrapolating on the abscissas the steepest portion (corresponding to the critical bias step) of the IV characteristics. Note that, except for very close to T_c, the critical current criterion is immaterial, since the IVs are discontinuous at I_c. Close to T_c, instead, the $V = V_{thres}$ criterion tends to overestimate the critical current, since a finite resistance (and thus a finite voltage) might appear at low bias due to uncompensated vortices that get depinned, or to vortex-antivortex depairing close to the Berezinskii-Kosterlitz-Thouless (BKT) transition. On the other hand, the alternative criterion (extrapolation on the current axis of the steepest portion of the IV) is difficult to apply near T_c since the step itself becomes highly smeared. In Supplementary Fig. **8c** we plotted only points where the IVs display a sufficiently well-defined voltage step. From the convergence of the open symbols on the T-axis ($I_c^\pm(T = T_c) = 0$ condition), we deduce a critical temperature $T_c = 4.3$ K. This value is slightly higher than that ($T = 3.68$ K) corresponding to the emergence of a finite zero-bias resistance. This discrepancy is probably due to the aforementioned dissipative processes occurring below the mean field T_c.

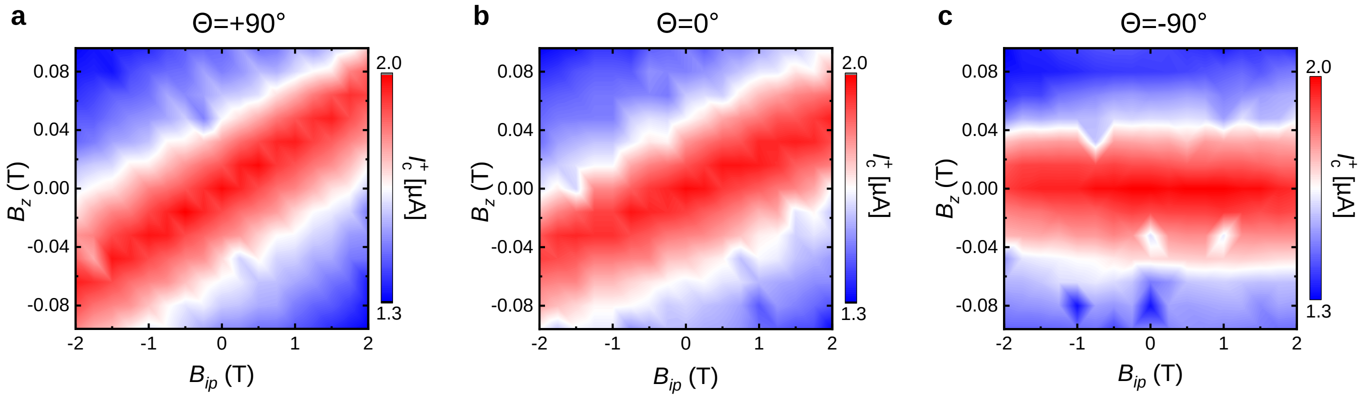

Supplementary Figure 7. **Critical current as a function of in- and out-of-plane field for different θ values. a**, Color plot of the positive critical current I_c^+ as a function of the *nominally applied* in-plane (B_{ip}) and out-of-plane (B_z) field, measured for an angle $\theta = 90°$ between current and $\vec{B}_{ip}$, c.f. Fig. 2**a** of the main text. The maximum of each $I_c^+(B_z)$ curve (vertical cut of the color plot) occurs at a field $B_{z,\mathrm{max}}$, which depends linearly on B_{ip}, indicating a misalignment as described in the text. **b** The same, measured for $\theta = 0°$, c.f. Fig. 2**b** of the main text. **c** The same, measured for $\theta = -90°$, c.f. Fig. 2**c** of the main text.

Supplementary Figure **8d** is the analogue of Supplementary Fig. **8c** for sample F. In this case from the convergence of the empty symbols onto the T-axis we deduce $T_c = 2.25$ K, which is very close to the temperature value ($T = 2.2$ K) where a finite resistance emerges.

In the main text, we have used as T_c values the ones deduced from the convergence of the $I_c^{\pm}(T)$ curves onto the temperature axis, i.e., the $I_c^{\pm}(T = T_c) = 0$ criterion. Therefore we assigned $T_c = 4.3$ K for sample D and $T_c = 2.25$ K for sample F.

SUPPLEMENTARY NOTE 8: OUTLIERS IN FIG. 2F OF THE MAIN TEXT

In Fig. 2**f** of the main text four points (three data points, $B_z = -48$, -64 and -80 mT for the curve $B_{ip} = 0$ T; one data point $B_z = -43$ mT for the curve $B_{ip} = -2$ T) were strong outliers. To reduce scatter we substituted the outliers with the corresponding points measured for the adjacent B_{ip} value (i.e., $B_{ip} = -0.25$ T instead of 0 T, $B_{ip} = -1.75$ T instead of -2 T). Supplementary Figure **9** shows the original data. The outliers are indicated by arrows. Outliers are also visible in the color plots in Fig. 2**a-c**. They might may conceivably originate from temperature instabilities during the measurements.

SUPPLEMENTARY REFERENCES

* nicola.paradiso@physik.uni-regensburg.de

[1] A subtraction of 170 nA to I_c^- corresponds to an addition of the same amount to $|I_c^-|$.

[2] N. Paradiso, A.-T. Nguyen, K. E. Kloss, and C. Strunk, Phase slip lines in superconducting few-layer NbSe2 crystals, 2D Materials **6**, 025039 (2019).

[3] X. Xi, Z. Wang, W. Zhao, J.-H. Park, K. T. Law, H. Berger, L. Forró, J. Shan, and K. F. Mak, Ising pairing in superconducting NbSe2 atomic layers, Nature Physics **12**, 139 (2015).

[4] L. M. Malard, T. V. Alencar, A. P. M. Barboza, K. F. Mak, and A. M. de Paula, Observation of intense second harmonic generation from MoS_2 atomic crystals, Phys. Rev. B **87**, 201401 (2013).

[5] Y. Li, Y. Rao, K. F. Mak, Y. You, S. Wang, C. R. Dean, and T. F. Heinz, Probing Symmetry Properties of Few-Layer MoS2 and h-BN by Optical Second-Harmonic Generation, Nano Letters **13**, 3329 (2013).

[6] K.-Q. Lin, S. Bange, and J. M. Lupton, Quantum interference in second-harmonic generation from monolayer WSe2, Nature Physics **15**, 242 (2019).

[7] F. Ando, Y. Miyasaka, T. Li, J. Ishizuka, T. Arakawa, Y. Shiota, T. Moriyama, Y. Yanase, and T. Ono, Observation of superconducting diode effect, Nature **584**, 373 (2020).

[8] C. Baumgartner, L. Fuchs, A. Costa, S. Reinhardt, S. Gronin, G. C. Gardner, T. Lindemann, M. J. Manfra, P. E. Faria Junior, D. Kochan, J. Fabian, N. Paradiso, and C. Strunk, Supercurrent rectification and magnetochiral effects in symmetric Josephson junctions, Nature Nanotechnology **17**, 39 (2022).

[9] J. J. He, Y. Tanaka, and N. Nagaosa, A phenomenological theory of superconductor diodes in presence of magnetochiral anisotropy (2021), arXiv:2106.03575v1 [cond-mat.supr-con].

[10] Y. Hou, F. Nichele, H. Chi, A. Lodesani, Y. Wu, M. F. Ritter, D. Z. Haxell, M. Davydova, S. Ilić, F. S. Bergeret, A. Kamra, L. Fu, P. A. Lee, and J. S. Moodera, Ubiquitous Superconducting Diode Effect in Superconductor Thin Films (2022), arXiv:2205.09276 [cond-mat.supr-con].

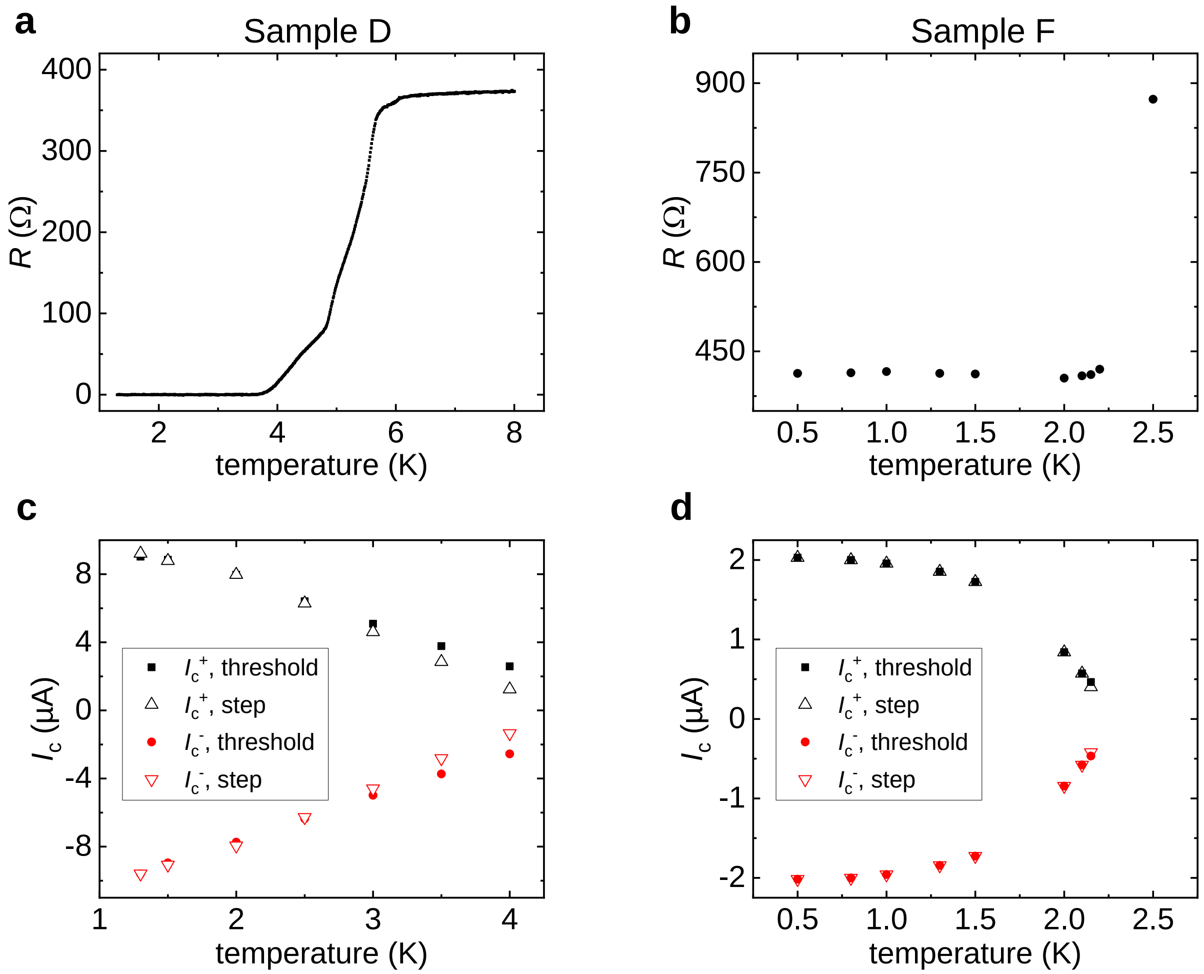

Supplementary Figure 8. $R(T)$ **and** $I_c^{\pm}(T)$ **for sample D and F. a**, Resistance versus temperature measured in a 4-terminal configuration on sample D. The first emergence of a detectable resistance above the noise occurs at $T = 3.68$ K. **b**, Resistance versus temperature measured in a 3-terminal configuration on sample F. The low temperature resistance (413 Ω) corresponds to the contact resistance. The first emergence of a detectable resistance above the noise occurs at $T = 2.2$ K. **c**, I_c^+ (black) and I_c^- (red) versus temperature for sample D. Full symbols refer to critical current values deduced from the threshold condition $V = V_{thr} \equiv 100$ µeV. Empty symbols refer to critical current values deduced by extrapolating the steepest portion of the IV to the abscissa axis. The latter points converge to $T_c = 4.3$ K. **d**, The same as in panel **c**, but for sample F. From the convergence of the empty symbols to the abscissa axis we deduce $T_c = 2.25$ K.

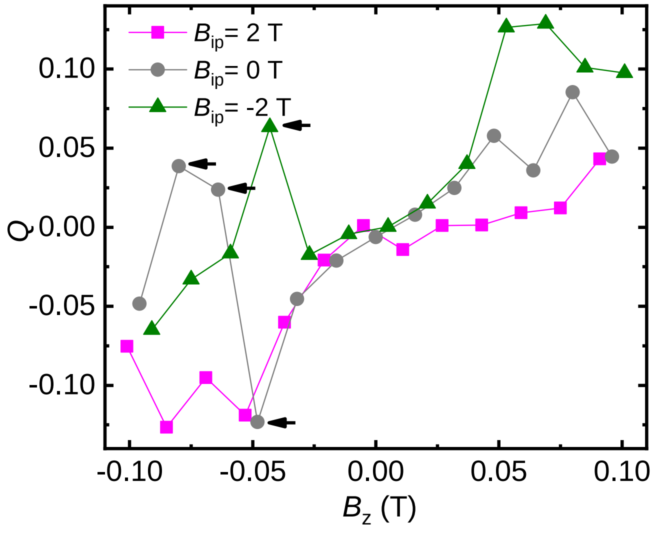

Supplementary Figure 9. **Figure 2f without outlier removal** The graph shows the data in Fig. 2**f** of the main text, without the outlier removal described therein. The arrows indicate the four points which were substituted in the main part of the article, see text.
